# The efficiency of clinical laboratories: the case of Kerman province

**DOI:** 10.1186/s12962-024-00564-x

**Published:** 2024-08-06

**Authors:** Zohreh Shaker, Zainab Shaker, Mohsen Barouni, Asma Sabermahani

**Affiliations:** 1 Health Human Resources Research Center, School of Health Management and Information Sciences, Shiraz University of Medical Sciences, Shiraz, Iran; 2 Faculty of Management and Medical Information Sciences, Kerman University of Medical Sciences, Kerman, Iran; 3 Department of Health Economics, Health Services Management Research Center, Institute for Futures Studies in Health, Kerman University of Medical Sciences, Kerman, Iran; 4 Health Foresight and Innovation Research Center, Institute for Futures Studies in Health, Kerman University of Medical Sciences, Kerman, Iran

**Keywords:** Clinical laboratories, Data envelopment analysis, Technical efficiency, Managerial efficiency, Variable returns to scale efficiency, Constant returns to scale

## Abstract

**Background:**

Medical diagnostic laboratories are an essential work environment that plays an important role in diagnosing, treating, and being sensitive to diseases. One way to evaluate laboratories’ performance is to calculate their efficiency. This study investigates the efficiency of laboratories that are related to health centers in the south of Iran.

**Methods:**

This study was conducted in 2021. The input numbers include: the number of technical personnel and the number of cell counters, and the output data includes: the scores obtained from the level 2 health laboratory evaluation list. And efficiency was calculated with DEAP software. The analysis is accomplished by the assumption of input-oriented.

**Findings:**

The efficiency of laboratories of Orzueeyeh and Ravar Cities had the highest efficiency with the assumption of variable returns to scale efficiency 1, and the model of all laboratories is the laboratory of Ravar City. The laboratories of Kuhbanan and Rabor cities had the lowest efficiency with the assumption of variable returns to scale efficiency of 0.859 and 0.899, respectively. The average scale efficiency, Variable returns to scale, and constant returns to scale for laboratories in the cities of Kerman province are 0.842, 0.943, and 0.895, respectively.

**Conclusions:**

To increase the efficiency of laboratories, significant resources and funds should be used, as well as few studies have been done on the efficiency of laboratories, which requires more attention.

## Introduction

A laboratory is typically a workspace for conducting experimental tests, measurements, quantity determination, quality control, detailed comparison of test methods, analysis, and identification of materials are performed. Medical laboratories are used to diagnose diseases and then prevent the spread of infectious diseases and fight against diseases. Today, according to new diagnostic methods, laboratories are one of the most important parts of the health system and account for approximately 1.7% of the total hospital costs. Laboratories are a very sensitive and important work environment due to the variety of work and the variety of specialists in different fields, so they need more attention and evaluation [1, 2].

Also, second-level laboratories are very important because they cover a large population. Iran's health system consists of three levels, level 2 includes units that can provide health and treatment services at a more specialized level than level 1, which includes health centers and city hospitals. In health centers, doctors provide health services along with experts, and these centers have facilities such as laboratory and radiology, dentistry, and midwifery. In Iran, the first and second-level units are located in the geographical boundaries of the cities [3].

Evaluation of these laboratories is one of the most important tasks that can be done to monitor the laboratory, and comparing the efficiency and quality control of public or private laboratories with standard (efficient) laboratories is one of the ways to improve and increase the safety of laboratories, which avoids errors in tests and reduces risks for personnel and patients [4].

There are various methods to evaluate the performance of laboratories and the resources allocated to them, one of which is efficiency studies. Efficiency is a management concept that has a long history in management science. Efficiency measures any performance that uses minimal inputs to get the maximum number of outputs [5]. Efficiency shows whether an organization has used its resources well in production at a point in time or not [6, 7].

There are different ways to calculate efficiency. One of these methods is the DEA method, which measures economic enterprises’ efficiency. Data Envelopment Analysis (DEA) is a technique that covers all the data. This method is a mathematical programming model to evaluate the efficiency of decision-making units (DMUs) that have multiple inputs and multiple outputs.

Initially, Farrell applied this method in 1957 to assess efficiency using a single input and output. Later, Charnes, Cooper, and Rhodes presented a model that included the ability to measure efficiency with multiple inputs and outputs [8].

This study employed the data envelopment analysis technique to gauge the efficiency of laboratories associated with the Kerman University of Medical Sciences. Considering the limited resources of the health sector, it is necessary to pay attention to increasing the efficiency of laboratories. Calculation and analysis of efficiency in cost control, optimal use of assets and property, and decision-making in the health and treatment sector will be of great help to managers and policymakers, so determining the optimal amount of inputs, and outputs and having a model for laboratories and determining the efficiency of laboratories is very important [5–7].

In 2014, Ketabi et al.’s study titled Efficiency Evaluation of Medical Diagnostic Laboratories Using Data Envelopment Analysis in Isfahan, Iran, examined the performance of 18 selected laboratories with the data envelopment analysis method, the output-oriented model, and a constant return to scale. They found that only 17% of the laboratories in Isfahan city have worked effectively [9].

In the study of Taheri et al., titled Efficiency of Clinical Laboratories Affiliated Shiraz University of Medical Sciences in 20 2015, he evaluated 10 selected laboratories. In this article, he used the Data Envelopment Analysis (DEA) method to determine the performance and found that most of the laboratories were highly efficient [10].

In 2020, in the study by Nejc Lamovšek et al. entitled Evaluation of Biomedical Laboratory Performance Optimization using the DEA Method, they performed an analysis of the performance of 20 biomedical laboratories in Slovenia and made a comparison with a "virtual" laboratory, i.e. Integration of laboratories in one organizational unit is chosen. The research results show that by evaluating the virtually merged laboratory, they determined that, under all three models, the virtual laboratory achieved 100% VRS efficiency [11].

Niloufar Ghafari Someh et al. in 2020 titled Performance Assessment of Medical Diagnostic Laboratories: A Network DEA approach. In this study, each medical diagnostic laboratory is considered a decision-making unit (DMU), and the NDEA model is used to calculate the efficiency of each medical diagnostic laboratory. The results show that three of the 22 considered laboratories were efficient [12].

Therefore, according to the investigations carried out so far, no study has been conducted regarding the efficiency of diagnostic laboratories in Kerman province. So, we decided to evaluate the efficiency of the diagnostic laboratories of Kerman University of Medical Sciences in 2021 by using the data envelopment analysis method.

## Methodology

This research is a descriptive-analytical and cross-sectional study that was conducted in Kerman province, the largest of the 31 provinces of Iran. This province is in the southeast of Iran and its center is the city of Kerman. In the 2015 census, the population increased to 3,164,718 people [13]. The study population is all the clinical laboratories of 9 cities, in Kerman province (Dadbin Kerman, Baft Center, Bardsir Center, Rabor Center, Shahr Babak Center, Zarand Center, Kuhbanan Center, Arzuiyeh Center, Ravar Center).

The sample size is equal to all government laboratories of Dadbin Kerman, Baft, Bardsir, Rabor Center, Shahr Babak, Zarand, Kuhbanan, Arzuiyeh, and Ravar, which are under the supervision of the Vice-Chancellor of Health of Kerman University of Medical Sciences. The inputs of the study included technical personnel and cell counters. The outputs include the score obtained from the Level 2 Health Laboratory System Quality Assessment Checklist of the Ministry of Health and Medical Education Iran [14].

The necessary information was through the checklist by referring to the laboratories (with the permission of the heads of the centers) all the collected data was related to 2021. This checklist has 138 questions and 11 sections, which include the following: laboratory personnel (10 questions), safety and hygiene in the laboratory (23 questions), laboratory equipment (12 questions), laboratory space and facilities (24 questions), process Before conducting the test (11 questions), the process of conducting the test (13 questions), the quality control of conducting the test (17 questions), the process after conducting the test (9 questions), its purchase and storage (11 questions), communication with other laboratories (5 questions), identifying and dealing with errors and cases of non-compliance (3 questions). In this checklist, there are 4 options to answer each question, including yes and no, and it does not require any corrective action or application. Each “yes” answer gets 2 points, each “no” answer gets zero points, and each question “requiring corrective action” gets 1 point. The answer “does not apply” is not included in the calculation and no points are assigned to it. The collected data were entered into Excel. Then, the average score obtained from the answers to the questions for each clinical laboratory is calculated. Finally, the score obtained by each laboratory is calculated as a percentage.

The analysis and calculation of economic, allocation, and technical efficiency types were accomplished by the assumptions of minimizing production factors (input-oriented) and variable returns to scale for evaluation and ranking of laboratory units.

It should be noted that the selection of inputs and outputs in this study was based on data accessibility. Also, the number of input and output and laboratories are considered based on the following formula [15] and condition:

$$\left(\text{number of inputs }+\text{ number of outputs}\right)\times 3=\text{ number of units}$$

To calculate the efficiency, the input data included the number of technical personnel and the number of cell counters, and the output data included the scores obtained by the laboratories of each city from the checklist. In this study, the technical efficiency of city laboratories was measured using data envelopment analysis (DEA). DEA is based on a series of optimizations using linear programming, which is also called a non-parametric method. In this method, the efficient frontier curve is created from a number of points determined by linear programming [7]. To calculate it, the weighted sum of the outputs is divided by the weighted sum of the inputs.

$$\max \,\theta_{j} = \frac{{\sum\limits_{{{\text{m}}\, = \,1}}^{M} {y_{{\text{m}}}^{j} \,u_{{\text{m}}}^{j} } }}{{\sum\limits_{{{\text{n}}\, = \,1}}^{N} {x_{n}^{j} \,v_{n}^{j} } }},$$

In this research, we used the data coverage analysis model with the assumption of constant return to scale (CRS) and the assumption of variable return to scale (VRS). In the CRS(Constant Returns to Scale) model, it is assumed that as the scale of production (resource level) increases, efficiency remains constant. In the VRS (Variable Returns to Scale) model, it is assumed that changes in the scale of production directly impact efficiency. In other words, increasing resources may result in either increased or decreased production [16].

Data analysis was done using DEAP software version 2.1. This software is specialized for calculating efficiency. Performance evaluation steps were carried out as follows:

1. Setting the list of desired inputs, by researchers
2. Setting the desired output list
3. Calculate the efficiency and determine the position of each unit
4. Determining efficient units

## Findings

The calculation of the efficiency of level 2 government laboratories, affiliated with the deputy health department of the cities of Kerman province (Dadbin Kerman, Baft, Bardsir, Rabor, Shahrbabak, Zarand, Kuhbanan, Orzueeyeh, and Ravar) for 2021, which is based on Data Envelopment Analysis method (DEA) was done. It should be noted that the health network laboratory and the hospital laboratory of small cities are considered as a subset and have been merged. The laboratories of Dadbin Kerman and Orzueeyeh cities had the highest input levels, and the lowest amount of input is related to Zarand and Ravar cities (Table 1).

**Table 1:** Inputs and output for the laboratory of each city

Row	Cities	The number of cells counters (Input 1)	The number of technical personnel (Input 2)	Score obtained (Output)
1	Dadbin Kerman	2	10	91
2	Baft	1	8	98
3	Bardser	1	6	98
4	Rabor	1	6	89.7
5	Shahrbabak	1	8	90
6	Zarand	1	5	91
7	Kuhbanan	1	9	85
8	Orzueeyeh	2	9	99
9	Ravar	1	5	99

The highest score (score obtained from the level 2 health laboratory evaluation checklist) is related to Orzueeyeh and Ravar cities with a score of 99% and the lowest score for Kuhbanan and Rabor cities with scores of 85 and 89.7% respectively.

The average efficiency of the scale, variable returns to scale (technical efficiency from VRS DEA), and constant return to scale (technical efficiency from CRS DEA) for the laboratories of the cities of Kerman province are 0.842 0.943 0.895 respectively.

**Fig. 1:**
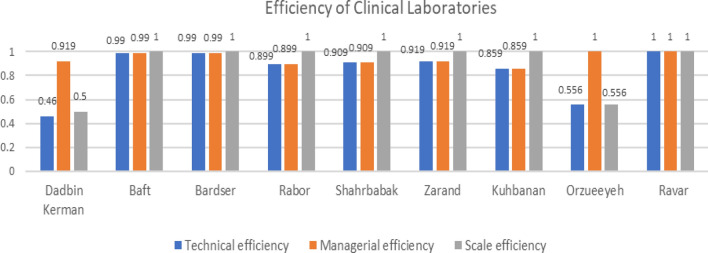
Efficiency of clinical laboratories for each city

The laboratories in Orzueeyeh and Ravar cities, assuming variable returns to scale at 1 (i.e., 100% efficient), can serve as a model for other cities, and the possibility of increasing the efficiency of the laboratories that were not efficient, without any cost It is possible. The laboratory of Ravar City is a model for all the laboratories of the investigated cities. The laboratories of Kuhbanan and Rabor counties with the assumption of variable returns to scale (given that Assuming constant returns to scale is impossible; the interpretation of the present study is based on the Variable returns to scale.) 0.859 and 0.899 respectively, the lowest efficiency had the Almost 22% of the laboratories were ineffective. The model of all the laboratories of the mentioned cities is the laboratory of Ravar city Variable and constant returns to scale for each city are given in Table 2 and Fig. 1.

**Table 2:** Variable and constant returns to scale for each city

Row	Cities	Technical efficiency	Managerial efficiency	Scale efficiency
1	Dadbin Kerman	0.46	0.919	0.5
2	Baft	0.99	0.99	1
3	Bardser	0.99	0.99	1
4	Rabor	0.899	0.899	1
5	Shahrbabak	0.909	0.909	1
6	Zarand	0.919	0.919	1
7	Kuhbanan	0.859	0.859	1
8	Orzueeyeh	0.556	1	0.556
9	Ravar	1	1	1

The findings indicate that, on average, reducing the number of personnel by 2.33 could lead to improved efficiency.

**Scheme 1:**
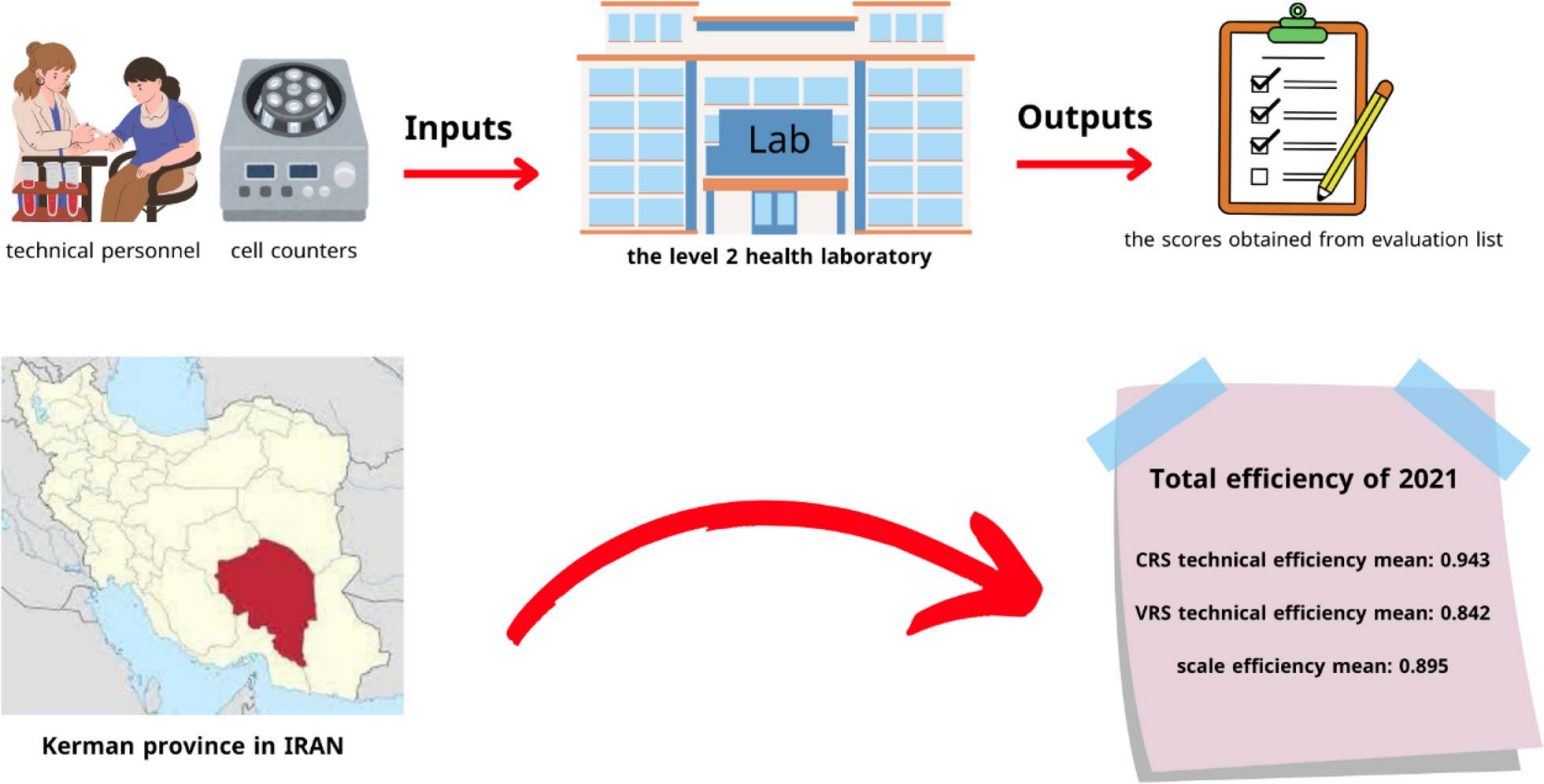
This research was conducted in Kerman province in Iran. The study population is all the clinical laboratories of 9 cities, in Kerman province. The input numbers include: the number of technical personnel and the number of cell counters, and the output data comprises: the scores obtained from the level 2 health laboratory evaluation list. Finally, efficiency was measured. The average scale efficiency, Variable returns to scale, and constant returns to scale for laboratories in the cities of Kerman province are 0.842, 0.943, and 0.895, respectively

For example, Dadbin Kerman laboratory, which has 10 personnel with an output of 91 (score obtained), can work more efficiently with 5 personnel, and also Kuhbanan and Orzueeyeh can increase their efficiency by reducing 4 personnel (Table 3) (see Scheme 1).

**Table 3:** slacks for inputs

Row	Input 1	Input 2
1	5	1
2	3	0
3	1	0
4	1	0
5	3	0
6	0	0
7	4	0
8	4	1
9	0	0
Mean	2.333	0.222

## Discussion

Due to the limited resources, the issue of reducing costs in the health system by improving performance has become an important concern. In response to this concern, numerous studies globally have aimed to measure the efficiency of different parts of the health system to help policymakers understand the current situation, which is the first step in any planning and decision-making.

Considering the importance of knowing about the performance and efficiency of laboratories, the conducted research showed almost good efficiency in most laboratories. The mean technical efficiency, calculated via the DEA method, was 84.2%, indicating that the existing laboratory units had a partial excess capacity and capacity improvement of technical efficiency of the clinical laboratories in those investigated hospitals was possible using the same level of inputs without costs increasing (up to 1.58%). To improve the efficiency of laboratories, it is necessary to pay attention to the training of employees on the correct use of equipment and test methods, and the improvement of the skills of employees. Also, upgrading laboratory equipment and devices to improve accuracy and efficiency and regular maintenance of equipment can be effective in increasing the efficiency of laboratories. In addition, identifying and dealing with errors and cases of non-compliance can help improve the performance of laboratories.

In general, most of the studies conducted are about evaluating the efficiency of hospitals. Few studies have been done on laboratory efficiency. Which makes it difficult to compare our results with those of other studies.

In a study by Ketabi et al. in 2014, they investigated the efficiency of 18 laboratories in Isfahan city, and only 17% of the laboratories were efficient with the assumption of constant return to scale and output-oriented model. The inputs in Ketabi et al.'s study differed from those in our research, which included: personnel wages, material costs, equipment costs, space and facilities costs. Outputs include the number of admissions, laboratory income, and compliance with required standards (such as physical standards of personnel, equipment and materials, space and facilities, safety process before and after testing, quality control, purchasing and storage, communication, and information) [9]. In our input-oriented study, the average efficiency, assuming constant returns to scale, was 0.895%, with only 11% of the laboratories (only Ravar) achieving efficiency.

The study by Taheri et al. in 2014, evaluated 10 selected laboratories from the diagnostic laboratories of hospitals affiliated to Shiraz University of Medical Sciences. In this article, they used the data envelopment analysis (DEA) method and found that 40% of the laboratories had increasing returns to scale efficiency and 60% of the laboratories had technical, managerial, and scale efficiency equal to one. The inputs of Taheri et al.'s study include the number of experts, experts, technicians, tools and equipment (such as a microscope, ELISA, cell counter, auto‐analyzer, centrifuge, and incubator), materials and solutions used, wages of experts, experts, and technicians, materials and solution prices, tools, and equipment prices. The output includes the number of hospitalized patients [8].

In the study of Alinjad et al., in 2013, the average economic efficiency of 22 laboratories affiliated to Urmia University of Medical Sciences was found to be 0.676. In this research, they used the Data Envelopment Analysis (DEA) method to calculate the efficiency and found that 76% of the laboratory units were not economically efficient. Inputs include: the number of specialists, specialists, technicians, tools and equipment, materials and solutions used, wages of specialists, experts and technicians, price of materials and solutions, and output includes the amount of laboratory income from admitted people [17]. In our study, 22% of the laboratories did not work.

In Lamovesk et al.’s 2020 study conducted in Slovenia, the data envelopment analysis method was utilized to calculate the efficiency of 20 primary-level healthcare laboratories. In this research, the input variables are labor (number of working hours), capital (number of biomedical analysts), and consumer goods (costs of laboratory materials). The output variables are the number of automatic and manual tests performed. Finally, the results were checked for three models and found that variable returns to scale in all three models were 100% and the constant return to scale was 92.94% [18].

## Conclusion

In light of the 22% inefficiency rate among the laboratories, it is imperative to intensify efforts towards enhancing the efficiency of this important department. Cities with lower scores ought to strive for improved results in the questionnaire, and due to the lack of studies in this field, more studies should be conducted in the future, and in future studies, efficiency can be measured with other inputs and outputs. (such as the number of common tests and the number of patients).

The most important factors in increasing the efficiency of laboratories are improving the knowledge and skills of human resources in using equipment, as well as identifying errors and how to deal with them. It should be noted that the improvement of capital resources can have an effect on the improvement of personnel performance. This type of study can improve testing processes and optimal management of the labor force and resources of clinical laboratories.

## Data Availability

No datasets were generated or analysed during the current study. Data supporting the article’s findings are available. We would like to express our gratitude to the Health Vice-Chancellor of Kerman University of Medical Sciences, who provided us with the necessary information about the laboratories of the province.
